# Blimp-1/PRDM1 is a critical regulator of Type III Interferon responses in mammary epithelial cells

**DOI:** 10.1038/s41598-017-18652-9

**Published:** 2018-01-10

**Authors:** Salah Elias, Elizabeth J. Robertson, Elizabeth K. Bikoff, Arne W. Mould

**Affiliations:** 1 Sir William Dunn School of Pathology, University of Oxford, South Parks Road, Oxford, OX1 3RE UK; 2 Biological Sciences, University of Southampton, Life Sciences Building 85, University Road, Southampton, SO17 1BJ UK

## Abstract

The transcriptional repressor Blimp-1 originally cloned as a silencer of type I interferon (IFN)-β gene expression controls cell fate decisions in multiple tissue contexts. Conditional inactivation in the mammary gland was recently shown to disrupt epithelial cell architecture. Here we report that Blimp-1 regulates expression of viral defense, IFN signaling and MHC class I pathways, and directly targets the transcriptional activator Stat1. Blimp-1 functional loss in 3D cultures of mammary epithelial cells (MECs) results in accumulation of dsRNA and expression of type III IFN-λ. Cultures treated with IFN lambda similarly display defective lumen formation. These results demonstrate that type III IFN-λ profoundly influences the behavior of MECs and identify Blimp-1 as a critical regulator of IFN signaling cascades.

## Introduction

The zinc finger transcriptional repressor Blimp-1 (PRDM1) originally identified as a post-inductive silencer of interferon beta (IFN-β) gene expression in virally infected cells^1^ and a critical regulator both necessary and sufficient for plasma cell terminal differentiation^2^ is now known to regulate gene expression profiles in multiple cell types^3^. By virtue of its ability to recruit chromatin modifiers such as HDAC1/2, LSD-1, and G9a, Blimp-1 governs epigenetic reprogramming required for germ cell specification in the early mouse embryo^4–6^. Blimp-1 null embryos die at around embryonic day 10.5 (E10.5) due to defective placental morphogenesis^4,7^. Loss of Blimp-1 disrupts specification of the spiral artery-associated trophoblast giant cell lineage (SpA-TGC’s) the crucially important cell subpopulation that invades and remodels maternal blood vessels^8,9^. Lineage tracing experiments in combination with expression profiling and single cell RNA-Seq analysis have defined the cell type specific transcriptional signature governing these specialized functional activities^8,9^.

In contrast in B and T lymphocytes, Blimp-1 function is not required at early stages during lineage commitment. Rather in B cells Blimp-1 directly silences expression of key transcription factors such as c-Myc, Pax5, and CIITA that maintain B cell identity to dramatically shift the developmental programme towards plasma cell terminal differentiation^10–12^. Recent studies have further characterized Blimp-1-dependent gene expression changes and chromatin remodeling at its transcriptional targets associated with plasma cell maturation^13^. Similarly Blimp-1 regulates cell fate choices made during differentiation of CD4^+^ T cell subsets, and controls the balance of cytolytic effectors vs the generation of memory CD8^+^ T cells^14–18^.

Recent experiments have shown that Blimp-1 governs postnatal reprogramming of intestinal enterocytes^19^. Blimp-1 function is required to prevent premature activation of the adult enterocyte biochemical signature^19^. ChIP-Seq analysis of E18.5 small intestine demonstrate Blimp-1 preferentially binds to promoter regions upstream of genes associated with metabolism^20^ and interestingly revealed a subset of highly conserved target sites, including the promoters of IFN-inducible components of the MHC class I antigen processing machinery such as Psmb8, Psmb10, Tapbp, and Erap1, that are also recognized by interferon regulatory factor (IRF) -1 a positive regulator of the MHC class I peptide loading pathway^20,21^. Thus Blimp-1 occupancy directly antagonizes IRF-1 to prevent premature activation of the MHC class I pathway in fetal enterocytes and maintain tolerance in the neonatal intestine in the first few weeks after birth during colonization of the intestinal tract by commensal microorganisms^20^.

Survival at these early stages depends on the highly specialized mammary glands that produce milk and enable the mother to feed her newborn offspring. The mammary epithelium contains two structurally and functionally distinct cell subpopulations: the outer myoepithelial/basal cells and the inner luminal cell population. Considerable progress has been directed towards understanding cell fate decisions during ductal morphogenesis, lumen formation, and alveologenesis. The functional contributions made by distinct mammary stem cell subpopulations, including both unipotent and bipotent progenitors have been extensively described in lineage tracing experiments^22^. Recent experiments demonstrate that Blimp-1 expression within a rare subset of luminal progenitors is up-regulated in response to pregnancy hormones and conditional inactivation results in defective mammary gland morphogenesis^23^. Strikingly Blimp-1 functional loss disrupts epithelial architecture and lumen formation both *in vivo* and in three-dimensional (3D) primary cell cultures^23^.

To further investigate the underlying causes of these tissue disturbances, here we performed transcriptional profiling experiments. To avoid studying possible contributions made by strongly Blimp-1-positive endothelial cells within this highly complex vascularized tissue *in vivo*, we exploited the inducible *ROSA26*:*CreERT2* allele to activate Cre-mediated Blimp-1 deletion via tamoxifen treatment of 3D mammary epithelial cell (MEC) cultures. As expected Blimp-1 deficient (cKO) MEC cultures display defective lumen formation and fail to establish apical-basal polarity. Surprisingly however functional annotation analysis of up-regulated genes identified highest enrichment scores for categories associated with innate immunity and IFN signaling pathways. This gene list substantially overlaps with those recently described as conserved Blimp-1 targets via ChIP-Seq analysis, including key components of the MHC class I peptide-loading pathway^20^. Additionally IFN-stimulated genes and pathway regulators including Usp18, Oligoadenylate synthetase (OAS) family members, and the key transcriptional activator Stat1, were identified here as direct Blimp-1 targets. Finally the present experiments demonstrate that Blimp-1 loss of function results in up-regulated expression of double stranded (ds)RNA and type III IFN lambda (IFN-λ). Moreover type III IFN-λ treatment of wild type MEC cultures causes defective lumen formation and maturation. These results demonstrate for the first time that the zinc finger transcriptional repressor Blimp-1 silences type III IFN expression in mammary epithelial cells and establish its actions as a global regulator of the IFN signaling cascade.

## Results

### Transcriptional profiling experiments demonstrate up-regulated expression of IFN signaling components

**Figure 1:**
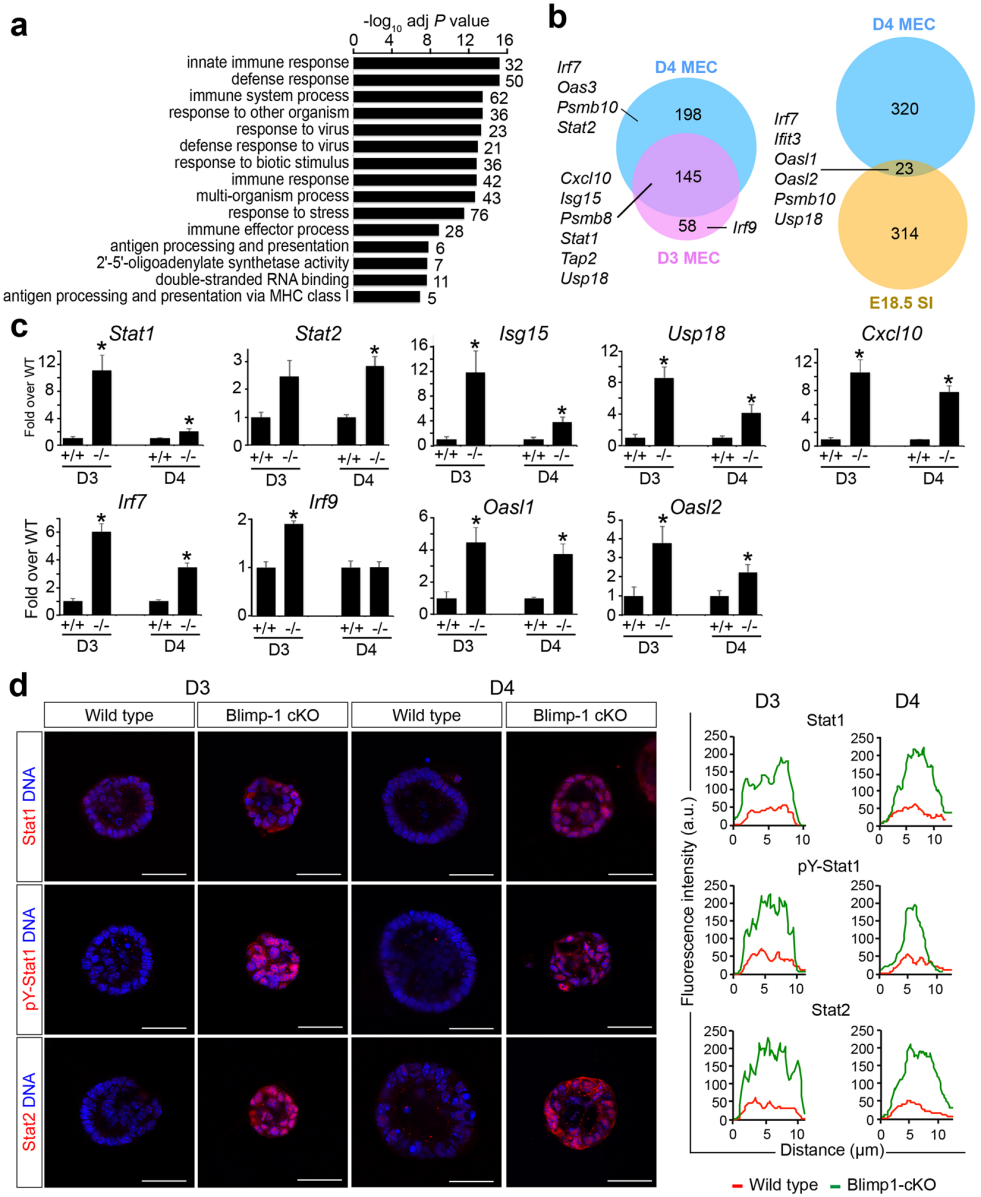
Transcriptional profiling experiments reveal up-regulated expression of anti-viral defense genes in Blimp-1 cKO mammary epithelial cell (MEC) cultures. (**a**) Significantly enriched GO terms among up-regulated genes in Day 4 Blimp-1 cKO MECs as determined by WebGestault. (**b**) Comparisons of up-regulated transcripts in Blimp-1 cKO MEC 3D cultures at Day 3 and Day 4 with those up-regulated in Blimp-1 cKO E18.5 small intestine identify components of viral defense, IFN signaling cascades and MHC class I antigen presentation pathways. (**c**) qPCR validation of transcriptional changes in cKO (−/−) relative to wild type (+/+). *P value < 0.05 (unpaired Student’s *t*-test). Data represents mean ± SEM of 5 samples per group. (**d**) Dramatically increased Stat1, pY-Stat1 and Stat2 expression in Blimp-1cKO 3D cultures. Representative line scan-analysis (representative fluorescence intensity, minimum 20 cells/genotype analyzed) confirms increased levels and nuclear localization of Stat1, pY-Stat1, and Stat2 in Blimp-1 cKO MECs. Scale bars: 50 μm.

To learn more about Blimp-1 functional contributions during mammary gland morphogenesis, we used the Illumina array platform to compare transcripts in wild type versus Blimp-1 cKO MEC cultures harvested at Day 3 (D3) and Day 4 (D4), when tissue disturbances become readily visible. The greatest differences in gene expression profiles were detectable at Day 4. The relatively few down-regulated genes (n = 138) in D4 Blimp-1 cKO MECs showed no significant enrichment for any GO annotated pathways or biological processes and were not investigated further (see Supplementary Table S1). In contrast, functional annotation clustering analysis of significantly up-regulated genes surprisingly revealed a striking enrichment for innate immunity, stress and viral response categories (Fig. 1a). Results summarized in Fig. 1b strongly suggest that Blimp-1 regulates expression of anti-viral effectors such as oligoadenylate synthetase (OAS) family members, IFN-stimulated genes (ISG’s) such as Usp18 and Isg15, IFN-inducible components of the MHC class I peptide-loading pathway, as well as the transcriptional activators Irf7, Stat1 and Stat2.

Additionally a subset of up-regulated transcripts overlapped with those previously identified in Blimp-1 cKO embryonic (E18.5) small intestine^19^. These expression changes were validated by quantitative PCR (qPCR, Fig. 1c), and additionally for Stat1 and Stat2 in cell staining experiments (Fig. 1d). Representative images shown in Fig. 1d demonstrate dramatically up-regulated and sustained predominantly nuclear Stat1, pY-Stat1 and Stat2 expression.

### Identification of overlapping Blimp-1 and Stat1 target genes

**Figure 2:**
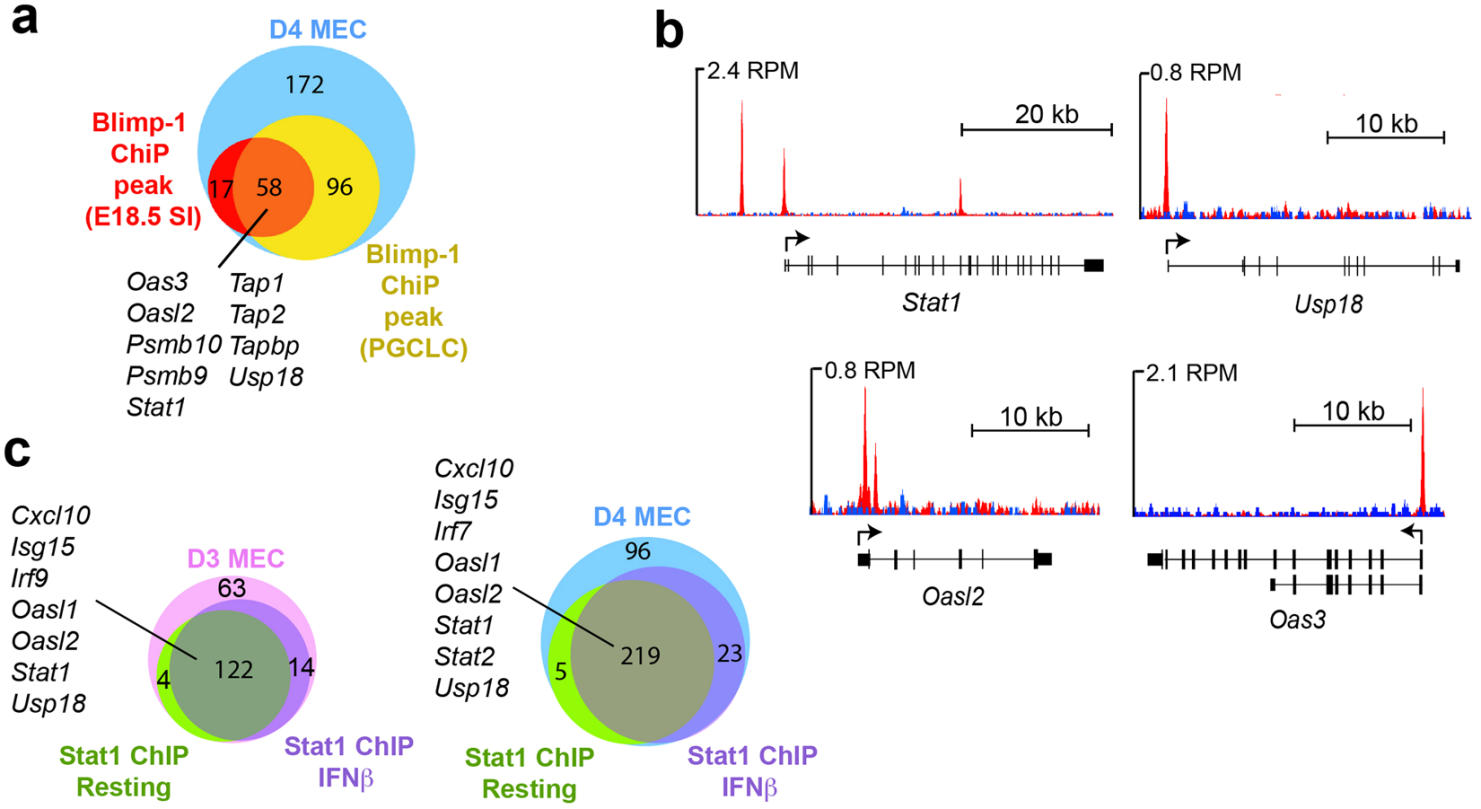
Comparisons with Blimp-1 and Stat1 ChIP-Seq datasets identifies direct target genes. (**a**) The subset of up-regulated genes with proximal Blimp-1 ChIP peaks includes *Stat1*, *Oas* family members, *Usp18*, and IFN-inducible components of the MHC class I antigen presentation pathway. (**b**) Blimp-1 binding at *Stat1*, *Usp18*, *Oasl2*, and *Oas3* genes (GSE66069). Positions of the TSS and direction of transcription are indicated by the arrows. **(c)** The majority of up-regulated genes contain proximal Stat1 ChIP-Seq peaks.

Comparisons of our list of up-regulated transcripts with the direct Blimp-1 target genes previously identified in ChIP-Seq experiments revealed substantial overlap (Fig. 2a). Moreover, there are Blimp-1 ChIP peaks present in the promoter regions of several IFN signaling pathway regulators - i.e., Stat1, Usp18, Oas3, and Oasl2 (Fig. 2b). Stat1 plays a central role in transcriptional activation of both type I and type II IFN responses^24,25^. Results above demonstrate increased Stat1 expression readily detectable at Day 3 the earliest time point examined (Fig. 1c). As shown in Fig. 2c, the vast majority of up-regulated genes identified here have been previously identified as Stat1 target genes, containing ChIP-Seq peaks constitutively occupied by Stat1 as well as those activated in response to type I IFN-β recognized by Stat1-Stat2 heterodimers together with IRF-9 - i.e., the so-called ISGF3 complex^24^. These observations suggest that dramatically increased and sustained Stat1 expression in the absence of Blimp-1 could potentially be responsible for activation of IFN-signaling genes.

Blimp-1 expression in the mammary gland is restricted to a rare subset of luminal progenitors, representing less than 1% of the total luminal cell population recovered from pregnant females^23^. The eGFP-tagged knock-in allele used previously for ChIP-Seq analysis of embryonic day (E) 18.5 small intestine is severely hypomorphic elsewhere^20^ and FACS sorting is problematic due to relatively weak eGFP fluorescence intensity even in plasma cells. For these reasons, we elected to perform qPCR validation of Blimp-1 occupancy at candidate target sites using transiently transfected CommaDβ cells, an established cell line that shares many functional and behavioral characteristics with bona fide mammary stem cells^26^, in combination with our expression construct encoding the C-terminal eGFP-tagged Blimp-1 fusion protein and the proven GFP mAb^27^. Results shown in Supplementary Figure S1 confirm Blimp-1 occupancy at key target sites in mammary epithelial cells.

### Blimp-1 inactivation results in accumulation of dsRNA

**Figure 3:**
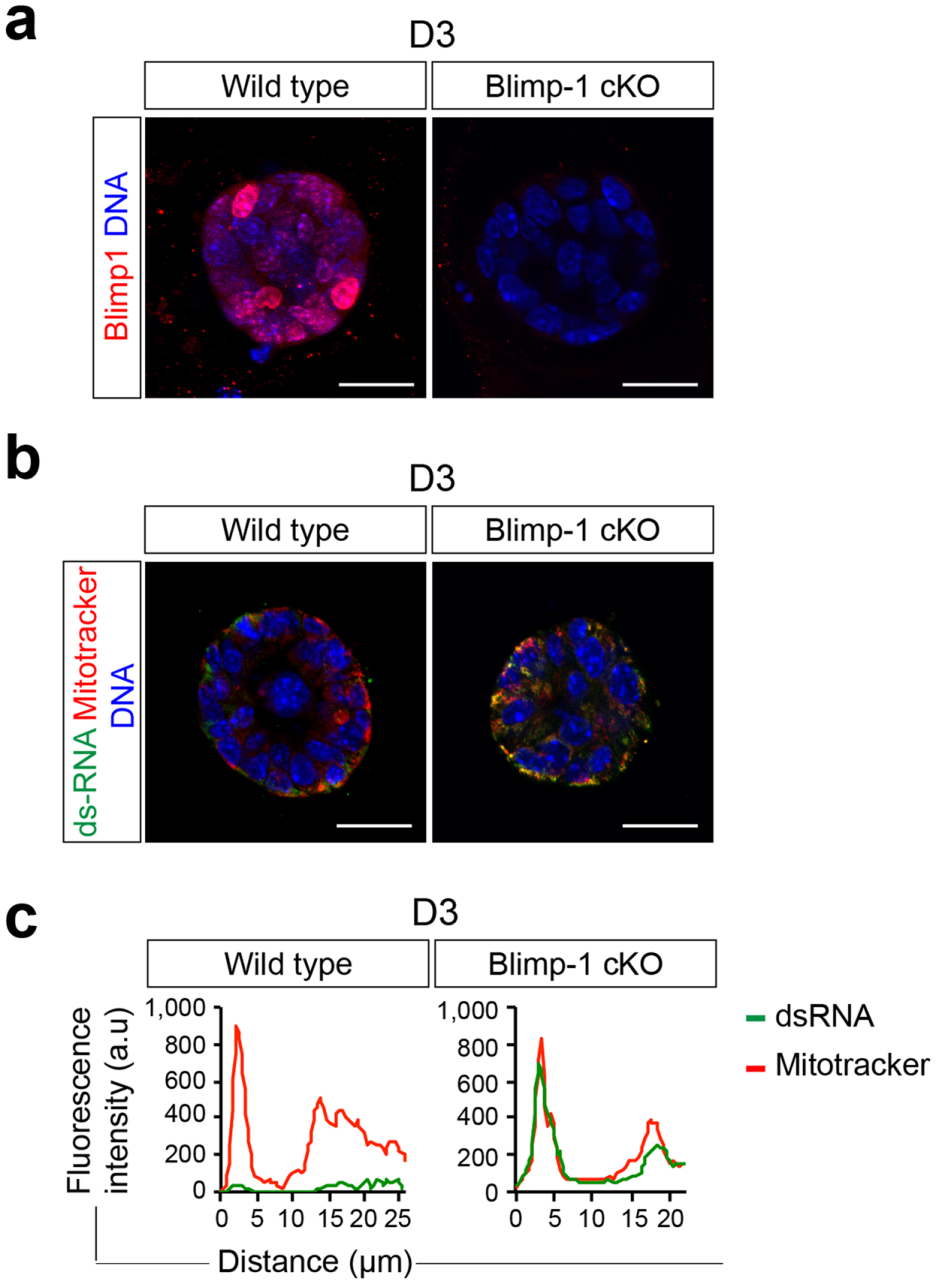
Blimp-1 inactivation in mammary epithelial cells results in accumulation of dsRNA. (**a**) Treatment with 4OHT activates Cre recombinase and eliminates Blimp-1 expression in mutant MEC 3D cultures. (**b**) Representative images of wild type and Blimp-1 cKO MEC 3D cultures co-stained for dsRNA using the J2 mAb and mitotracker to localize mitochondria. (**c**) Representative line scan-analysis (relative fluorescence intensity, minimum 20 cells/genotype analyzed) confirms increased levels of dsRNA and mitochondrial co-localization. Scale bars: 50 μm.

Recent studies demonstrate that cytosolic recognition of viral RNAs by helicases such as RIG-1 leads to the activation of IFN signaling responses^28,29^. Our transcriptional profiling experiments shown above reveal that Blimp-1 conditional loss causes up-regulated expression of several dsRNA binding proteins including the DEXD/H box helicases Ddx60, Dhx58 (LGP2), and RIG-1 (Ddx58) (Fig. 1a). However, these were not identified as direct target genes. Interestingly, recent studies demonstrate increased expression of dsRNA is associated with global epigenetic re-programming in human colorectal cancer cells exposed to DNA-demethylating agents^30^. We wondered whether Blimp-1 functional loss might similarly activate viral mimicry pathways upstream of IFN signaling responses. To test this possibility, next we used the well characterized J2 monoclonal antibody that specifically recognizes dsRNA greater than 40 bp in length^31^ to evaluate possible Blimp-1–dependent changes in dsRNA expression levels. As shown in Fig. 3, Blimp-1 cKO Day 3 MEC cultures display increased levels of cytoplasmic dsRNA closely associated with the mitotracker mitochondrial marker.

### Expression of type III IFN-λ by Blimp-1 cKO mammary epithelial cells

**Figure 4:**
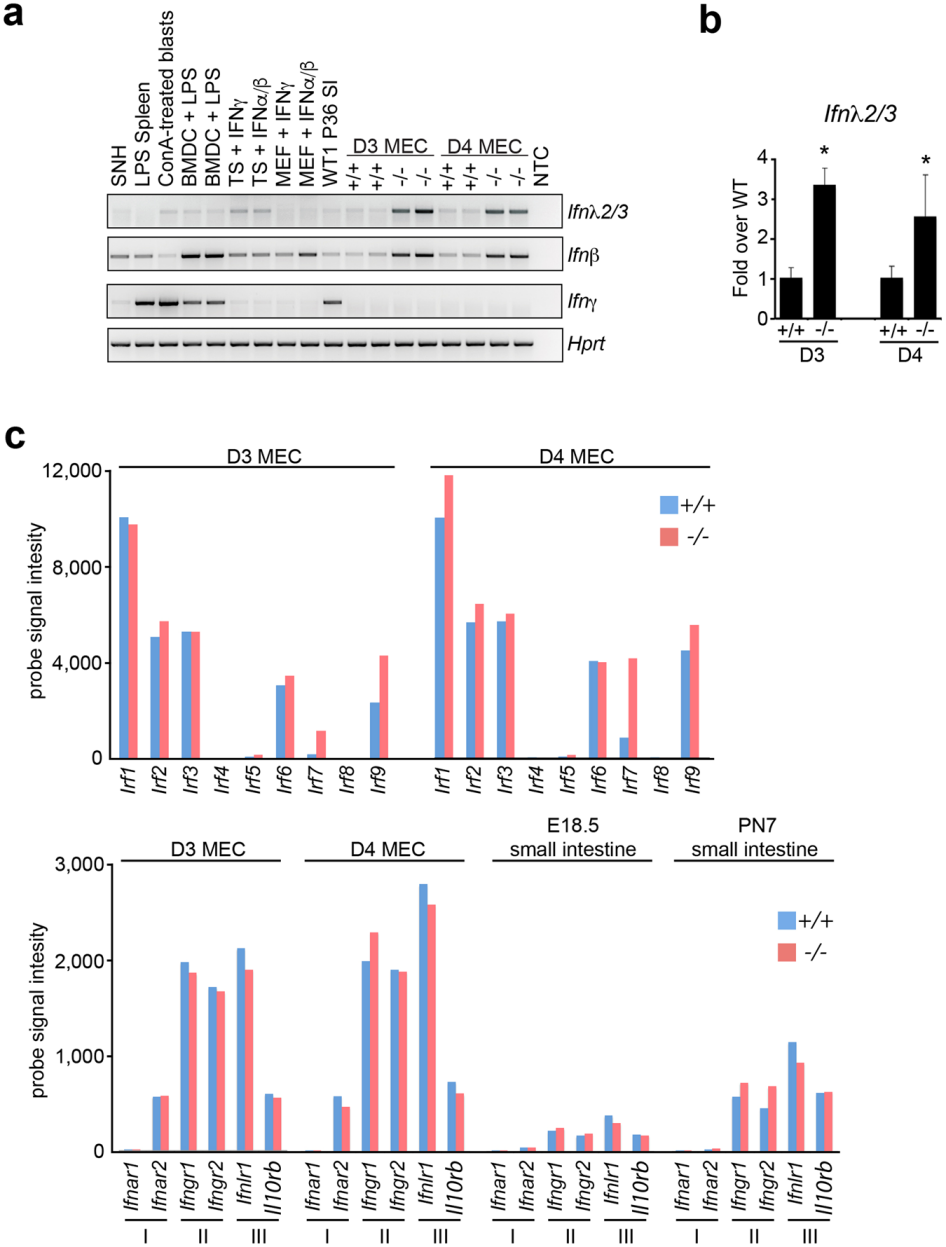
Blimp-1 functional loss activates type III IFN-λ expression in mammary epithelial cells. (**a**) RT-PCR analysis demonstrates induction of Type III λ and Type I β but not Type II γ IFN transcripts in Blimp-1 cKO MEC 3D cultures. Abbreviations: +/+, Wild type; −/−, Blimp-1 cKO; BMDC, bone marrow-derived dendritic cells; LPS, Lipopolysaccharide; ConA, Concanavalin; A SNH, STO cell line; MEF, mouse embryonic fibroblasts; TS, trophoblast stem cell; P36 SI, postnatal day 36 small intestine. Cropped PCR gel images shown. For uncropped gel images see Supplementary Figure S2. (**b**) qPCR validation of increased type III IFN-λ2/3 transcription. *P value < 0.05 (unpaired Student’s t-test). Data represents mean ± SEM of 4 samples per group. (**c**) Mammary epithelial cell 3D cultures robustly express multiple Irf family members and the type III IFN lambda receptor and as determined by average microarray probe signal intensity. Blimp-1 cKO MEC 3D cultures express increased levels of Irf7 (see also Supplementary Table S1). For comparison, microarray probe signals from embryonic (E) day 18.5 and postnatal (P) day 7 wild type and Blimp-1 cKO small intestine datasets corresponding to NCBI GEO accession numbers GSE29658 and GSE30556 respectively, are shown.

Recent experiments demonstrate that type III IFN-λ via its unique receptor complex selectively activates anti-viral defense pathways in epithelial cells^32,33^. Considerable evidence demonstrates that type III IFN-λ plays a crucial protective role at mucosal barrier surfaces lining the respiratory, gastro-intestinal, and reproductive tracts^32,33^. Blimp-1 was previously shown to silence IFN-λ1 expression in human lung epithelial cells^34^. However in mice IFN-λ1 is a pseudogene. To test whether Blimp-1 regulates type III IFN-λ expression in mouse mammary epithelial cells, here we performed RT-PCR experiments using primers designed to detect IFN-λ2/3 transcripts. As shown in Fig. 4a and b, expression of type III IFN-λ2/3 and type I IFN-β was dramatically up-regulated in Blimp-1 cKO MEC cultures. In contrast, there was no evidence for expression of type II IFN-γ by these highly organized epithelial cell cultures.

Overlapping functional roles played by individual IRF family members have been intensely investigated^21^. Irf-3 and Irf-7 have been shown to induce Type I and Type III IFN expression in virally infected cells^35^. Interestingly we found here that Irf-1, 2, 3, and 6 are constitutively expressed by mammary epithelial cells whereas Blimp-1 inactivation leads to increased expression of Irf-7 and Irf-9 (Fig. 4c). Additionally IFN-λ receptor complexes were robustly expressed by MEC 3D cultures (Fig. 4c) suggesting that Type III IFN-λ could potentially influence the behavior of mammary epithelial cells.

### Treatment with Type III IFN lambda disrupts epithelial cell polarity

**Figure 5:**
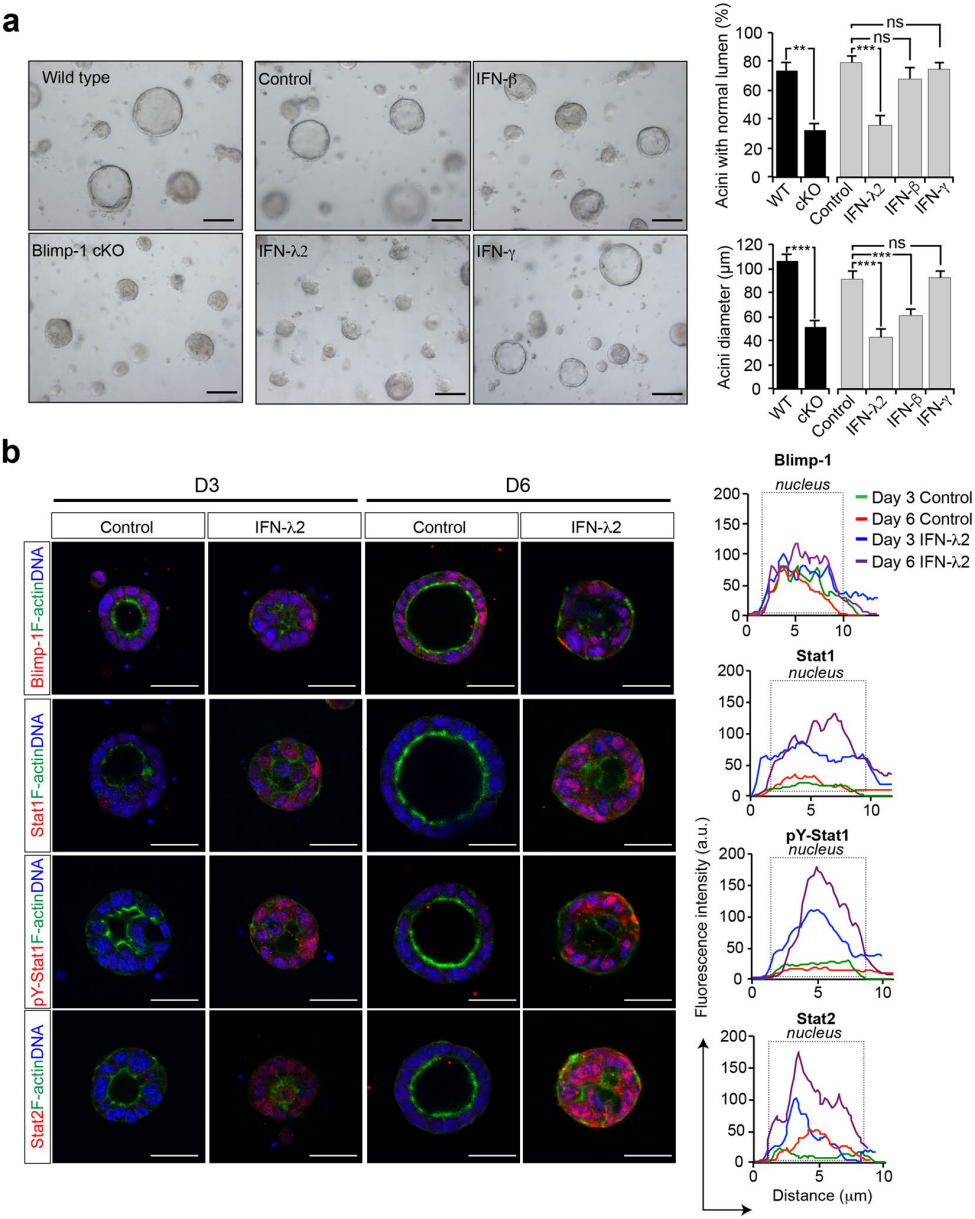
Disturbances caused by IFNλ treatment closely resemble those observed in Blimp-1 cKO 3D MEC cultures. (**a**) Phase contrast images of D6 MEC 3D cultures treated with recombinant type III IFN-λ2 reveal defective lumen formation and maturation. No noticeable changes were observed in the presence of Type II IFN-γ, whereas treatment with Type I IFN-β resulted in an intermediate phenotype. Bar graphs compare the percentages of acini with normal lumen and average acini diameters observed for Blimp-1 cKO vs wild type MEC cultures treated with recombinant IFNs. **P value < 0.01, ***P value < 0.001, relative to control (cKO vs WT - unpaired Student’s *t*-test, inter-treatment comparisons - ANOVA followed by Tukey post-hoc test). Data represents mean ± SEM of 282 (WT), 305 (cKO), 134 (Control), 112 (IFN-λ2), 145 (IFN-β) and 127 (IFN-γ) acini (normal lumen formation) and 104 (WT), 114 (cKO), 134 (Control), 112 (IFN-λ2), 145 (IFN-β) and 127 (IFN-γ) acini (diameter analysis). (**b**) Immunostaining demonstrates dramatically increased Stat1, pY-Stat1, and Stat2 expression induced by treatment with type III IFN-λ. Representative line scan-analysis (relative fluorescence intensity, in arbitrary units, a.u., minimum 20 cells per group analyzed). Scale bars: 50 μm.

To directly test this possibility, recombinant IFNs were added at Day 0 to MEC 3D cultures. As shown in Fig. 5a, IFN-λ treatment resulted in dramatic morphological changes including the collapsed acini phenotype, as well as dramatically up-regulated Stat1, pY-Stat1, and Stat2 expression (Fig. 5b). Moreover treatment with IFN-λ resulted in up-regulated gene expression profiles as described above for Blimp-1 cKO MEC 3D cultures (Supplementary Fig. S3). In contrast we failed to observe any noticeable effect in the presence of type II IFN-γ, whereas treatment with type I IFN-β was associated with an intermediate phenotype (Fig. 5a and Supplementary Fig. S4). These results demonstrate for the first time that as for diseases associated with excessive type I IFN activities^36^, that type III IFN-λ also has potentially detrimental effects. Thus it appears that Blimp-1 normally functions to restrain type III IFN-λ signaling and maintains the ability of mammary epithelial cells to differentiate normally and undergo ductal morphogenesis.

## Discussion

The ability of Blimp-1 to bind specifically to the PRD1 element of the human IFN-β gene promoter and silence expression was characterized nearly three decades ago^1^. We now understand that Blimp-1 plays essential roles governing cell fate decisions in a wide variety of tissue contexts dependent not only on target site selection but also its ability to recruit co-repressor complexes required to silence gene expression^3^. Recent experiments exploiting powerful high throughput methodologies have provided detailed insights into its functional activities guiding lineage commitment and terminal differentiation in specialized cell populations^6,9,13^. Early studies revealed that the Blimp-1 consensus binding motif closely resembles the IRF-E sequence element recognized during activation of IFN responses. Ironically at present, relatively little is known about Blimp-1-dependent regulation of innate immunity or IFN signaling pathways.

The transcriptional networks and signaling pathways controlling dynamic developmental changes observed in the mammary gland during puberty, pregnancy, lactation, and involution, have been intensely investigated^22^. Our recent experiments demonstrate that Blimp-1 expression in a rare subset of luminal progenitors plays an essential role in mammary gland development^23^. Blimp-1 regulates proliferation and luminal cell maturation during puberty and pregnancy. Conditional inactivation causes defective epithelial cell polarity and disrupts milk secretion^23^. To learn more about Blimp-1 functional activities in the mammary gland, here we performed transcriptional profiling experiments.

Surprisingly up-regulated genes in Blimp-1 cKO MEC 3D cultures were greatly enriched for key regulators of viral defense, innate immunity, and IFN signaling pathways. This result was entirely unexpected since only healthy animals were used in our experiments. Besides protective roles played by IFN signaling activated during viral infections, constitutive expression of type I IFN at low levels in the absence of viral infection is also known to be required for the maintenance of tissue homeostasis under diverse circumstances. The importance of so-called tonic signaling in hematopoietic stem cells^37^, thymic epithelial cell development^38^ and immune cell function^39^ has been previously characterized. Recent experiments suggest that Type I IFN could potentially regulate functional properties of mammary stem cells^40^. Adaptive immune responses including CD4^+^ T cell production of type II IFN-γ have been shown to contribute to mammary gland organogenesis^41^. In contrast here to avoid potential contributions made by lymphocytes, endothelial cells, macrophages, and local stromal elements, we exploited 3D mammary epithelial cell cultures where the formation of self-organized acini structures recapitulates those normally observed during mammary gland organogenesis. Under these circumstances, we found that Blimp-1 inactivation resulted in up-regulated expression of type I and type III IFNs, but there was no evidence for expression of Type II IFN-γ.

The present experiments demonstrate for the first time in the mammary epithelium that Blimp-1 directly silences expression of IFN signaling pathway regulators including Oas family members, the ubiquitin-specific peptidase Usp18, and importantly the transcriptional activator Stat1 that universally drives gene expression changes upstream of Type I, Type II, as well as Type III IFN signaling cascades. The balance between Type I vs Type II target site selection by Stat1 reflects its phosphorylation status and distinct transcriptional partnerships^24^. Additionally unphosphorylated Stat1 has been shown to selectively regulate target gene expression^25,42^. For example, its association with IRF-1 activates constitutive LMP2 gene expression^43^. Here we report in the absence of Blimp-1 that up-regulated genes substantially overlap with those activated by Stat1. Thus, by virtue of its ability to directly antagonize Stat1 expression, Blimp-1 potentially functions as a global regulator of IFN signaling pathways.

Since its discovery in 2003, Type III IFN-λ has been shown to selectively provide anti-viral protection in epithelial cells, particularly those present at the mucosal surfaces lining the respiratory and gastrointestinal tracts^32,33^. Recent experiments suggested that Blimp-1 is a transcriptional repressor of IFN-λ1 expression in virally infected human airway epithelial cells^34^. However, the type III IFN genes display considerable divergence across evolution and human IFN-λ1 corresponds to a pseudogene in mice^44^. Interestingly however ChIP on chip experiments identified highly conserved Blimp-1 binding sites adjacent to the human IFN-λ2 promoter^45^ and Blimp-1 knockdown increased levels of IFN-λ2/3 transcripts in cultured human airway epithelium^34^. Consistent with the idea that Blimp-1 recognizes these highly conserved ISRE sites, here we observed up-regulated IFN-λ2/3 expression in Blimp-1 cKO MEC cultures. Thus, combinatorial regulatory motifs controlling type III IFN gene expression closely resemble those governing type I IFN-β expression^34,35^.

The simplest scenario is that Blimp-1 occupancy at ISRE/PRD1 sites directly silences IFN-λ2/3 gene expression. Unfortunately however the eGFP-tagged Blimp-1 knock-in allele successfully used for ChIP-Seq analysis in E18.5 intestinal epithelium is only weakly expressed in the mammary gland in a rare subpopulation of luminal progenitors. Occupancy at selected target sites was validated in qPCR experiments using transiently transfected and FACS-sorted CommaDβ cells enriched for expression of the eGFP-tagged Blimp-1 fusion protein. However we found no evidence for significant binding at predicted sites upstream of the IFN-λ2/3 gene promoters. Unfortunately ectopic Blimp-1 over-expression is toxic excluding analysis of stably transfected cell lines and limiting our ability to explore Blimp-1 occupancy at multiple time points. The present results clearly demonstrate that Blimp-1 function is required to prevent type III IFN-λ expression in mammary epithelial cells. However, additional work will be needed to characterize its role in assembly of higher order complexes governing chromatin accessibility and transcriptional output at this locus.

Recent studies implicate a functional relationship between epithelial cell polarity and IFN-λ expression from peroxisomes^46^. Cytoplasmic sensors such as RIG-1 (Ddx-58) that recognize viral RNA trigger membrane bound signaling proteins such as MAVS that in turn activate assembly of transcriptional complexes driving IFN-stimulated gene expression. In contrast to these disturbances downstream of viral entry and replication, the striking morphological changes described here associated with up-regulated IFN-λ expression are simply caused by Blimp-1 inactivation. Interestingly Blimp-1 cKO also resulted in accumulation of dsRNA recognized by the J2 monoclonal antibody. Thus, activation of IFN signaling responses could simply be due to the presence of dsRNA.

The J2 mAb recognizes dsRNA greater than 40 bp in length but its fine specificity remains ill defined. An extensive literature has described diverse host mechanisms responsible for controlling expression of endogenous retroviral sequences scattered across the genome. It is tempting to speculate that as for many other zinc finger proteins, Blimp-1 may directly silence ERV transcription in a sequence specific manner. However mapping ChIP seq peaks to repetitive elements requires the application of sophisticated bioinformatics strategies. An important long term goal is to characterize dsRNAs that accumulate in the absence of Blimp-1 and test this attractive model.

The present results help to resolve the paradoxical finding previously described in our recent studies, namely how could Blimp-1 inactivation in rare progenitor cell population cause such dramatic widespread phenotypic changes throughout the entire epithelial cell population^23^. We now understand in the absence of Blimp-1 that type III IFN-λ secretion by rare luminal progenitors acting in a paracrine fashion has the ability to influence the behavior of neighboring cells. Our results demonstrate for the first time that type III IFN-λ not only activates anti-viral responses in mucosal epithelial cells, but also in mammary epithelial cells. While the role played by type III IFNs in innate immune protection at mucosal barrier surfaces has been extensively investigated, relatively little is known about viral infections in the mammary epithelium or how this impacts on neonatal health. It will be important to learn more about protective and potentially negative effects mediated by type III IFN-λ.

## Methods

### Animals

Female C57BL/6 mice (8–10 weeks of age) were used as the wild type strain. For inducible Blimp-1 gene deletion in mammary epithelial cell (MEC) cultures, *Prdm1* ^*BEH/*+^ and *Prdm1* ^*CA/CA*^ mice^23^ were crossed with the *ROSA26:Cre* ^*ERT2*^ line^47^ to generate *Prdm1* ^*BEH/CA*^ *;ROSA26:Cre* ^*ERT2*^ females. Mice were genotyped by PCR as described in the original reports. All animal experiments were performed in accordance with Home Office regulations and were approved by the University of Oxford Local Ethical Committee.

### Cell lines and primary MEC 3D cultures

CommaDβ cells (a generous gift from Marina Glukhova, Institut Curie) were maintained in DMEM/F12 medium supplemented with B27 (GIBCO), 20 ng/mL epidermal growth factor (EGF, Invitrogen), 20 ng/mL basic fibroblast growth factor (bFGF, GIBCO) and 10 μg/mL insulin (Sigma-Aldrich) as previously described^26^. Primary MECs were collected from 15.5- and 16.5-day pregnant mice and cultured as described^48^. Cells (2 × 10^4^) were seeded onto growth factor reduced Matrigel (120 μl per well in 24 well plates, BD Bioscience, or 8-well Lab-Tek II chamber slides, Thermo Fisher Scientific) to form acini and cultured in growth media [DMEM/F12 medium (Life Technologies) containing 5 μg/ml insulin, 1 μg/ml hydrocortisone (Sigma-Aldrich), 3 ng/ml EGF, 10% FBS, 50 U/ml penicillin/streptomycin, 0.25 μg/ml fungizone and 50 μg/ml gentamycin)] supplemented with 2% growth factor reduced Matrigel. Cultures were fed every 48 hrs and grown for 3–6 days. Cre-mediated deletion of Blimp-1 in primary MEC cultures (hereinafter referred to as Blimp-1 cKO) was achieved by collecting MECs from *Prdm1* ^BEH/CA^;*ROSA26:Cre* ^*ERT2*^ mice and treating with 1/1000 dilution of 4-Hydroxytamoxifen (4-OHT) dissolved in ethanol (100 nM final concentration) for 24 hrs. As controls, MECs from WT mice were similarly treated with 4-OHT. In some experiments, MitoTracker Red CMXRos (Invitrogen) dissolved in MEC growth media (200 nM final concentration) was added at day 2 of culture for 45 min at 37 °C. Recombinant mouse IFN-β (500 U/mL, PBL Assay Science), IFNγ (5 ng/ml, PeproTech) or IFN-λ2 (20 ng/ml, PeproTech) was added on day 0 of culture.

### Immunofluorescence

For immunofluorescence staining, acini were fixed with 4% PFA in PBS and permeabilized with 0.5% Triton X-100 in PBS. Fixed cells were blocked with 10% normal goat serum/2% BSA in PBS for 2 hrs at RT, and incubated with Blimp-1, Stat1, pStat1 (Tyr701), Stat2, or dsRNA (J2) antibodies (see Supplementary Table S2 for antibody dilutions and source) in block overnight at 4 °C. Primary antibodies were detected by incubation with an appropriate secondary Alexa Fluor-conjugated antibody (Molecular probes). F-Actin was detected using Phalloidin-Alexa Fluor 633 (Invitrogen). Acini with F-actin staining at the apical surface of cells surrounding a single lumen were identified as acini with normal lumens. For all immunostaining, slides were mounted in DAPI-containing Fluoroshield. The pictures were captured with an inverted Olympus FV1200 laser scanning confocal microscope equipped with X40 and X60 oil-immersion objective. Z stack steps were of 1 μm. Images were analyzed with ImageJ.

### RNA extraction, qRT-PCR and Microarray profiling

Total RNA from five independent MEC 3D cultures each from *Prdm1* ^*BEH/CA*^;*ROSA26*:*Cre* ^*ERT2*^ and wild type treated with tamoxifen on day 0 and harvested on Day 3 and 4 was isolated using an RNeasy Mini kit (Qiagen) with on column DNase treatment according manufacturer’s protocol. Microarray transcriptional profiling experiments were performed using triplicate RNA samples of each genotype at both Day 3 and 4 as previously described using Illumina Mouse WG-6 v2 Expression BeadChips^8^. Differential probe expression was determined following rank-invariant normalization using the Illumina custom error model option of Gene Expression Analysis Module V1.6.0 of GenomeStudio V2009 (Illumina) with Benjamini and Hochberg false discovery rate. Probes with significant different expression (1.5 fold and differential score ≥ 13, equivalent to P ≤ 0.05) were analyzed by WebGestalt using default parameters (http://www.webgestalt.org/option.php). qPCR analysis was performed on 5 RNA samples for each genotype at day 3 and 4. First-strand cDNA was reverse transcribed from RNA (1 μg) using Superscript III (Invitrogen, UK) according to manufacturer’s protocol and qPCR performed using QuantiTech SYBR Green master mix (Q2040143; Qiagen) on a Rotor-Gene Q (Qiagen). Relative gene expression was calculated using the ΔΔCt method in comparison with *Hprt* as the reference. Reverse transcription-PCR (RT-PCR) was performed using the OneStep RT-PCR kit (Qiagen) according to manufacturer’s protocol. Primer sequences are provided in Supplementary Table S3.

### Published ChIP-Seq dataset analysis

ChIP-Seq datasets corresponding to NCBI GEO accession numbers GSE66069, GSE60204 and GSE33913 were used to identify Blimp-1^6,20^ and Stat1^24^ genomic binding sites. ChIP-peaks were functionally annotated with GREAT version 3.0.0^49^, using the basal extension rule linking peaks to the nearest transcription start site (TSS) ± 100 kb.

### qPCR-ChIP

CommaDβ cells were transiently transfected with a plasmid expression construct encoding C-terminal eGFP-tagged Blimp-1 fusion protein^27^ using Lipofectamine 2000 (Invitrogen) according to the manufacturer’s protocol. GFP positive cells, sorted by FACS at 24–48 hours post transfection were cross-linked with 1% formaldehyde in growth media for 20 min at RT and subsequently processed for ChIP using 10 μg of mouse anti-GFP IgG2a (clone 3E6, A11120; Invitrogen), as described previously^50^. Non-transfected CommDβ cells were used as negative controls. Eluted DNA samples were recovered using ChIP DNA Clean and Concentrator column kit (Zymo Research) and qPCR analysis of quadruplicate ChIP and input samples performed using QuantiTech SYBR Green master mix (Q2040143; Qiagen) on a Rotor-Gene Q (Qiagen). Primers were designed to amplify 100–200 bp regions central to Blimp-1 binding sites identified in ChIP-Seq dataset GSE66069. Selected genes included *c-Myc, Prdm1*, *Stat1* (both TSS and 5’ of TSS ChIP-Seq peak regions –see Fig. 2b), *Oasl2 and Usp18*. A non-enriched ChIP-Seq region in the 3’UTR of the *Prdm1* gene was used as a negative control^20^. Primer sequences are provided in Supplementary Table S3. Fold enrichment of ChIP over input was determined relative to a standard curve generated from log-diluted sheared genomic DNA.

### Statistical analysis

Statistical analyses were performed using two-tailed unpaired *t*-test for wild type versus Blimp-1 cKO comparisons and ANOVA followed by Tukey post-hoc test to compare between treatment conditions (Microsoft Excel and GraphPad Prism 7.0.). *P* values ≤0.05 were considered significant.

### Data availability

The microarray data has been deposited in NCBI GEO with the accession number GSE100747.

## Electronic supplementary material

Dataset 1

Supplementary information
