# Supplementary material for: Blimp-1/PRDM1 is a critical regulator of Type III Interferon responses in mammary epithelial cells
Source: Sci Rep. 2018 Jan 10;8:237. doi: 10.1038/s41598-017-18652-9 (PMC5762727; doi:10.1038/s41598-017-18652-9)
Supplement: Supplementary file 2 — Supplementary information [file 41598_2017_18652_MOESM2_ESM.pdf]

Supplementary Information

Blimp-1/PRDM1 is a master regulator of Type III Interferon responses in mammary epithelial cells

Salah Elias, Elizabeth J. Robertson, Elizabeth K. Bikoff, and Arne W. Mould

Supplementary Table S2. List of antibodies. Related to Figures 1, 2, 3 and 5.

Antibody	Supplier	Cat No	Host Species	Dilution
anti-Blimp-1	Santa Cruz Biotechnology	sc-130917	Rat monoclonal	1 in 500
Phalloidin-Alexa Fluor 633	Invitrogen/MP	A22284		1 in 100
anti-Stat1 p84/p91 (E-23)	Santa Cruz Biotechnology	sc-346	Rabbit polyclonal	1 in 200
Anti-pStat1 (Tyr701) (58D6)	Cell Signaling	9167	Rabbit monoclonal	1 in 100
Anti-STAT2 [EP1814Y]	Abcam	ab134192	Rabbit monoclonal	1 in 200
Anti-dsRNA (J2)	SCICONS	10010200	Mouse monoclonal	1 in 200

Supplementary Table S3. Primers used in this study. Related to Figures 1 and 4 and Supplementary Figures S1 to S3.

Gene	Purpose	Forward primer	Reverse primer
Prdm1 Exon1B	ChIP qPCR	ACTGCACATACCTGGCACTC	TCTTCCTCTTGCACTGGTTG
Prdm1 3'UTR	ChIP qPCR	AATACAGGACCGAACCACCC	GGAGTGCAGGAATTAATGTCGT
Stat1 5' TSS peak	ChIP qPCR	CGGACAGGCTGTGGGAAA	GTGCTTTCTGGGAACTCAGC
Stat1 promoter peak	ChIP qPCR	AATCTCTGCCGCTGATTGG	GAGCTTTGACAGACTCGGC
Oasl2	ChIP qPCR	CTCCTGTTCCCTTCTGCCTT	GCATGGAAAGAAGAAAAGCGC
Usp18	ChIP qPCR	GCCTGAGTTTCGCTTTCCTT	GAGTGTCTGCTGTCCCCTAG
Myc	ChIP qPCR	TGCGGTGACTGATATACGCA	ACCATTTTCTCTTGCTCGCG
Prdm1	RT-qPCR	GGCTCCACTACCCTTATCCTG	TCCTTTTGGAGGGATTGGAGTC
Stat1	RT-qPCR	TGCTTCCCATGTCTCCAGAG	CGCCAGAGAGAAATTCGTGT
Stat2	RT-qPCR	GCTCTACGGTGTGCTTGTG	TGTCCCCTGTCCCAGTTATTATT
Isg15	RT-qPCR	TCCTTAATTCCAGGGGACCT	TAAGACCGTCCTGGAGCACT
Usp18	RT-qPCR	TTCCCTCAGAGCTTGGATTTC	CCGGATGTAGGCACAGTAATG
Cxcl10	RT-qPCR	TTGAAATCATCCCTGCGAGC	TGGTCTTAGATTCCGGATTCAGA
Irf7	RT-qPCR	TGATCCTGGTGAAGCTGGAG	GGGATTCTGAGTCAAGGCCA
Irf9	RT-qPCR	CCTCTTTGTTCAGCGCCTTT	CCTGGAAGTACTGGGCCAAA
Oasl1	RT-qPCR	CAGACCCCACCAACAATGTG	CTGCACGGTCACCTGGATAT
Oasl2	RT-qPCR	CATCCTAGACCCAGCTGACC	TCTCACCTGAACATCCCTCG
Hprt	RT-qPCR and One-Step RT-PCR	GCTGGTGAAAAGGACCTCT	CACAGGACTAGAACACCTGC
IFNλ2/3	RT-qPCR and One-Step RT-PCR	TGGGAGTGAATGTGGCTCAG	AGCTGCAGGCCTTCAAAAAG
IFNβ	One-Step RT-PCR	CTACAGGGCGGACTTCAAGA	AGTGGAGAGCAGTTGAGGAC
IFNγ	One-Step RT-PCR	TCCTGCAGAGCCAGATTATCT	ATCAGCAGCGACTCCTTTTC

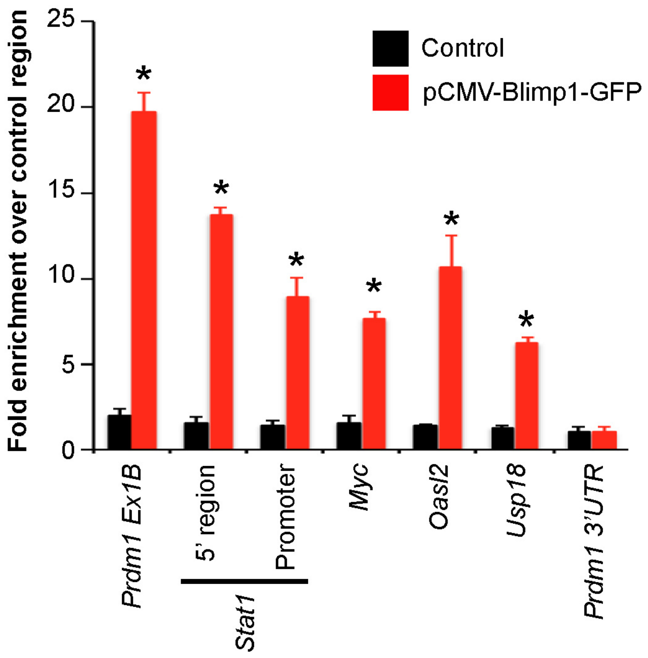

Supplementary Figure S1. qPCR validation of Blimp-1 target genes. Related to Figure 2 and Table S3. Transiently transfected CommaDβ mammary epithelial cells expressing eGFP-tagged Blimp-1 fusion protein were processed for ChIP using the anti-GFP monoclonal antibody.

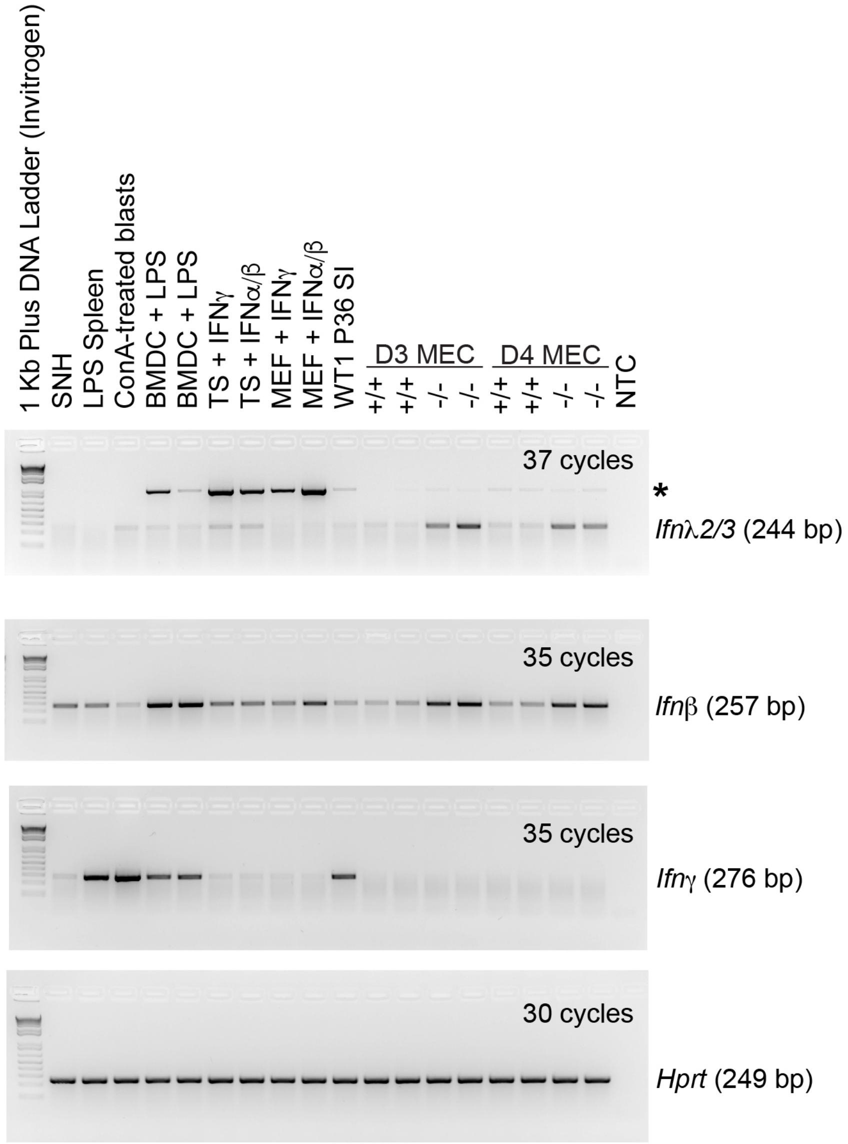

Supplementary Figure S2. Uncropped RT-PCR gel images related to Figure 4.
PCR cycle numbers for each transcript are indicated. *A higher molecular weight *IFNλ2/3* PCR amplicon (approximately 950 bp) is also observed in some positive control samples due to the amplification of residual levels of gDNA at the higher PCR cycle number.

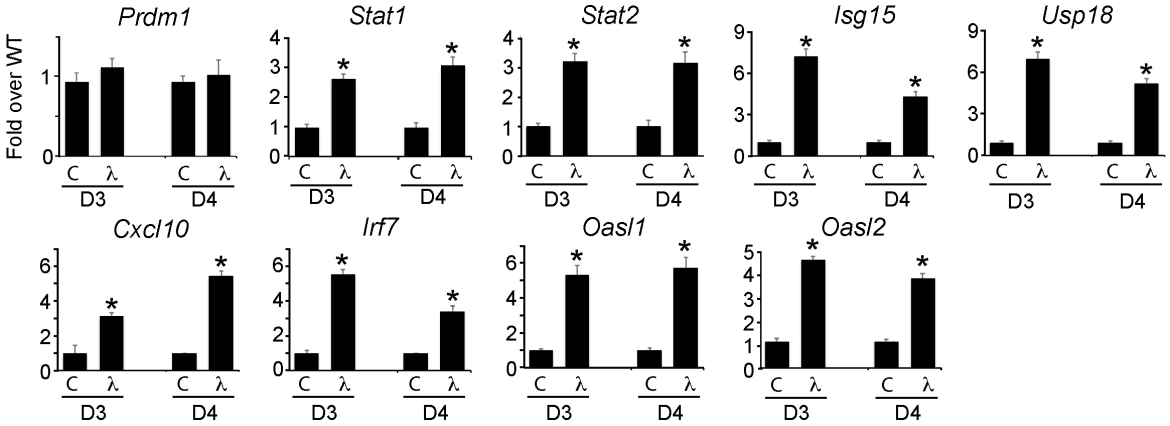

Supplementary Figure S3. **qPCR analysis validates up-regulated expression of IFN signaling genes caused by treatment with type III IFN lambda.** Related to Figure 5. *P value < 0.05 (unpaired Student's *t*-test). Data represents mean ± SEM of 4 samples each for control (C) and IFN lambda (λ) -treated groups.

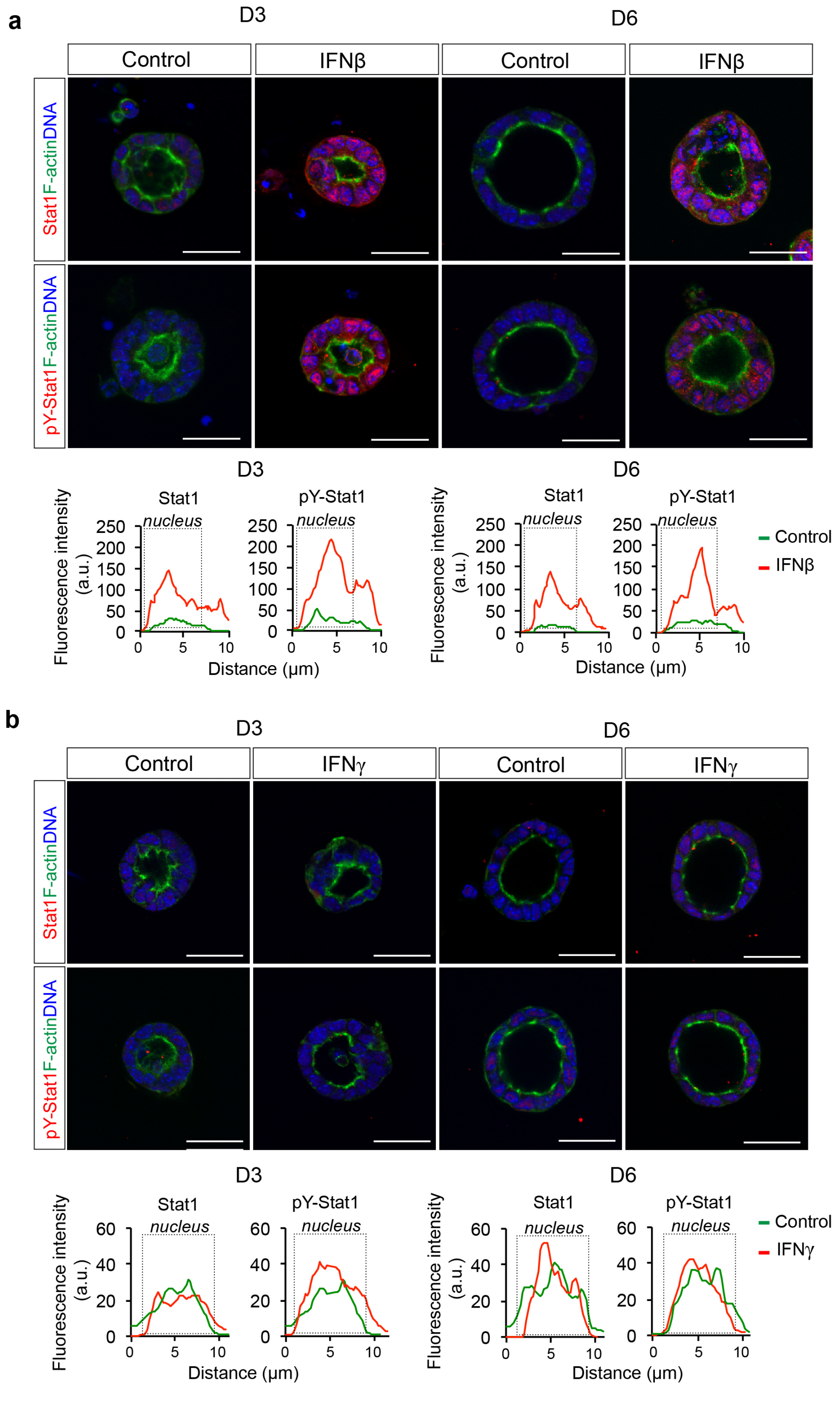

Supplementary Figure S4. **Increased levels of Stat1 and pY-Stat1 induced by treatment with type I IFN beta but not type II IFN gamma**. Related to Figure 5. MEC 3D cultures treated with (**a**) IFN beta and (**b**) IFN gamma and stained for Stat1 and pY-Stat1 by immunofluorescence. Representative line scan-analysis (fluorescence intensity in arbitrary units, a.u., minimum 20 cells/group analyzed). Scale bars: 50 μm.
